# Exercise – not as hot as you think

**DOI:** 10.1113/JP278528

**Published:** 2019-08-06

**Authors:** Peter Aldiss

**Affiliations:** ^1^ Institute of Metabolism & Systems Research University of Birmingham Birmingham UK

**Keywords:** Exercise, Adipose tissue, Thermoneutrality, Housing temperature

It is becoming increasingly evident that housing temperature is a critical factor to consider when conducting murine research. Indeed, it was suggested as early as 1939 that the thermoneutral zone of the mouse was between 30 and 33°C and not the 20–22°C in which they are now routinely studied. This is important because the general physiology, metabolism and immunity of the mouse at these two temperatures is strikingly different. Indeed, if given the option, mice will choose to spend a large proportion of their time (55% *vs*. 10% at 20°C) at these higher temperatures (Gordon, 2017). The current thinking therefore is that studying mice in their thermoneutral zone better represents human physiology as we also choose to spend most of our time at thermoneutrality. And, whilst there is ongoing debate about how warm mice should be, research groups have utilised thermoneutral housing to better understand the pathophysiology of tumour growth, atherosclerosis and non‐alcoholic fatty liver disease.

Brown adipose tissue (BAT) is a key thermogenic organ in mice which produces heat following the activation of uncoupling protein 1 (UCP1) on the inner mitochondrial membrane. At standard housing temperatures BAT is hyperactive, and thermogenic ‘beige’ adipocytes are present in the inguinal white adipose tissue (IWAT). At lower temperatures, heat production from these thermogenic adipocytes contributes significantly to basal metabolic rate as mice defend their body temperature in a cold environment and further cold exposure improves a myriad of metabolic and pathophysiological traits. At thermoneutrality, however, the thermogenic programme is suppressed and the beige characteristic of IWAT is less evident with subsequent cold exposure having a much smaller effect on whole body metabolism.

In recent years it has been shown that exercise training induces the ‘browning’ of IWAT and that this induction of thermogenic adipocytes could be one of the ways in which exercise improves metabolic health. However, ‘browning’ is not consistently evident in humans following exercise though the reason(s) for this discrepancy are unclear. In this issue of *The Journal of Physiology* McKie *et al*. (2019) show that thermoneutral housing attenuates the ‘browning’ and mitochondrial biogenesis seen in IWAT of male mice following exercise training. Thermoneutral housing alone decreased the mass of this characteristically beige depot by 22% and, whilst voluntary wheel running increased markers of mitochondrial biogenesis at 22°C, these effects were blunted at thermoneutrality. Whilst McKie *et al*. suggest that these environmental differences contribute, at least in part, to the lack of ‘browning’ seen in humans following exercise it must be noted that induction of thermogenic genes in adipose tissue is typically associated with reductions in adiposity. The same is seen here and it is feasible, as previously suggested, that ‘browning’ is secondary to a reduction in adiposity and an increased sensation of peripheral cold whereby ‘browning’ acts as a compensatory response (Aldiss *et al*. 2018). It would be interesting to see if ‘browning’ is evident in a model of exercise training where adiposity is maintained such as the Siberian hamster. McKie *et al*. also show that BAT mass is decreased following exercise training. The opposite is true in a number of murine studies and increased BAT mass is a characteristic of athletes or trained humans which has previously been attributed to a ‘whitening’ of the depot in animal models. A caveat of these findings is that no functional tests were carried out on BAT or IWAT to determine whether mitochondrial respiration differed between groups. This will be an important addition to future studies looking at the impact of housing temperature and should consider the two types of mitochondria (i.e. cytoplasmic and peri‐droplet) in BAT.

Of particular interest here is the finding that animals at thermoneutrality run ∼2 km more per week than mice at standard housing temperatures. And, whilst this contradicts other recent data showing animals run less at thermoneutrality (Raun *et al*. 2019; Takahashi *et al*. 2019) McKie *et al*. suggest this increase in running capacity can be explained by reduced thermal and metabolic stress, as evidenced by reduced levels of corticosterone at thermoneutrality.There was no difference in skeletal muscle mitochondrial proteins or hepatic glycogen between exercise trained groups suggesting neither of these factors contributed to increased exercise performance. However, consideration should also be given to the range of other cardiovascular and physiological parameters which are altered by housing temperature. For instance, heart mass, heart rate (∼25 beats/min for every 1°C decrease below 30°C) and mean arterial pressure (∼2 mmHg for every 1°C decrease below 30°C) are increased at 20°C. Animals also spend much more of their time awake at standard housing temperature and it has been previously suggested that everything we know comes from ‘sleep‐deprived and chronically hypertensive animals’. Add in cold‐stressed, hypermetabolic and hyperphagic and you begin to understand why translation of these studies to the clinic is low. Nevertheless, any one, or a combination of these factors, may underlie the increased running capacity.

The work from McKie *et al*. comes at the same time as two pre‐prints (Aldiss *et al*. 2019; Raun *et al*. 2019). The latter of these also shows blunted adaptations to exercise at thermoneutrality including reduced insulin action and protein expression in muscle and thermogenic genes in adipose tissue and adaptations to the microbiome. Our work, whilst only investigating the effects of exercise training in obese animals raised from weaning at thermoneutrality shows that UCP1 mRNA is not expressed in IWAT and cannot be induced with training. Collectively, these works highlight the importance of thermoneutrality in the interpretation and translation of rodent exercise studies. What we currently know is built on the physiology of the cold‐stressed mouse and much work is needed to understand rodent physiology at thermoneutrality. Fortunately, this area is hot right now.

## Additional information

### Competing interests

None declared.

### Author contributions

Sole author.

### Funding

This work was supported by a Wellcome Trust Senior Fellowship (GGL‐104612/Z/14/Z).
